# Effects of* Morinda officinalis* Polysaccharide on Experimental Varicocele Rats

**DOI:** 10.1155/2016/5365291

**Published:** 2016-12-20

**Authors:** Lihong Zhang, Xiaozhen Zhao, Feng Wang, Qing Lin, Wei Wang

**Affiliations:** ^1^Department of Human Anatomy, Histology and Embryology, School of Basic Medical Sciences, Fujian Medical University, No. 1 Xueyuan Road, Minhou County, Fuzhou, Fujian 350108, China; ^2^Research Center for Neurobiology, School of Basic Medical Sciences, Fujian Medical University, No. 1 Xueyuan Road, Minhou County, Fuzhou, Fujian 350108, China

## Abstract

*Morinda officinalis* is a traditional Chinese herbal medicine, which has been used to tonify the kidney and strengthen yang for a long time in China. In this study, the effects of* M. officinalis* Polysaccharide (MOP) on experimental varicocele adolescent rats were investigated. The result showed that varicocele destroyed the structure of the seminiferous epithelium and decreased the TJ protein expression (Occludin, Claudin-11, and ZO-1), testosterone (T) concentration in the left testicular tissue and serum, and serum levels of inhibin B (INHB), while increasing the levels of cytokines (TGF-*β*3 and TNF-*α*) in the left testicular tissue, as well as serum levels of gonadotropin-releasing hormone (GnRH), follicle-stimulating hormone (FSH), luteinizing hormone (LH), and antisperm antibody (AsAb). MOP repaired the damaged seminiferous epithelium and TJ and reduced the levels of cytokines (TGF-*β*3 and TNF-*α*) as well as serum levels of GnRH, FSH, LH, and AsAb, while upregulating TJ protein expression, T level in the left testicular tissue and serum, and serum INHB levels. In summary, we conclude that MOP promotes spermatogenesis and counteracts the varicocele-induced damage to the seminiferous epithelium and TJ, probably via decreasing cytokines (TGF-*β*3 and TNF-*α*) levels and regulating the abnormal sex hormones levels in experimental varicocele rats.

## 1. Introduction

*Morinda officinalis* (Bajitian in Chinese), the dried rhizome of plant* Morinda officinalis F.C.How*, has been used as a traditional Chinese medicine to tonify the kidney, strengthen yang, and treat impotence, menstrual disorders, rheumatoid arthritis, immune deficiency, and osteoporosis for a long time [1].* M. officinalis* Polysaccharide (MOP), the main bioactive chemical component of* M. officinalis* (accounting for 10.55–35% of the total weight) of different origins in China [2, 3] has many biological activities, including antitumor, antisenility, and antiviral activities as well as enhancing immunity and scavenging free radical. Our previous study indicated that* M. officinalis* aqueous extract (containing most of the MOP) was more effective than the alcohol extract in promoting spermatogenesis and encouraging the plerosis of injured reproductive organs [4, 5], indicating that MOP may be the main effective component that tonifies the kidney and strengthens yang in* M. officinalis*.

Tight junctions in the testis, the most important composition of the blood-testis barrier (BTB), are formed by three families of transmembrane proteins (Occludin, Claudin, and JAM) and a cytoplasmic protein (ZO) [6]. Tight junction not only establishes a specialized microenvironment for spermatogenesis but also permits the regular passage of preleptotene and leptotene spermatocytes across the BTB for further development [7]. Many signaling molecules and transcription factors, especially hormones and cytokines, are involved in TJ dynamics [8]. For example, testosterone (T) can significantly increase the Sertoli cell-TJ permeability barrier in vitro [9] and stimulate the expression of Occludin [10]. Meanwhile, testosterone at 1 × 10^−7 ^mol L^−1^ (similar to the testicular level) can protect the Sertoli cell-TJ-permeability barrier from CdCl_2_-induced destruction in vitro [10]. Furthermore, follicle-stimulating hormone (FSH) inhibits the expression of Claudin-11 in vitro [11] and gonadotropin-releasing hormone (GnRH) can regulate the organization of TJ protein in vivo [12]. The cytokines (e.g., TGF-*β*3, TNF-*α*, and IL-6) produced by Sertoli and germ cells in the testis downregulate TJ protein levels [11, 13–17]. For example, TGF-*β*3 can disturb the Sertoli cell-TJ barrier, possibly via its effects on inhibiting the synthesis of TJ proteins (Occludin, Claudin-11, and ZO-1) during TJ assembly in vivo [18]. Thus, interactions between hormones, cytokines, and TJ proteins play a crucial role in TJ assembly and regulation of TJ dynamics. Changes in both hormone and cytokine levels caused by diseases or toxicity may disturb the TJ-permeability barrier and affect TJ functionality via effects on TJ protein expression and distribution.

Varicocele can be described as an abnormal vascular dilation of the pampiniform plexus, which is the most identifiable common cause of male infertility [19]. Most varicoceles occur on the left side, which may be induced by the higher probability of left spermatic venous valves absence [20] and the slower velocity of blood flow of the left renal vein (the left spermatic vein injects into the left renal in a right angle) than the inferior vena cava (the right spermatic vein drains into the inferior vena cava in a sharp angle) [21]. The prevalence of varicocele is as high as 15–20% in the general population; meanwhile, it is related to almost 30–40% of male infertility cases, and the upright walking posture of humans may lead to the high incidence of this disease [22–24]. Despite the long history associated with varicocele and extensive research on animal models and human cases, the pathophysiology and the specific contribution of varicocele to male infertility are still controversial. So far, there are several conceivable mechanisms involved in the pathophysiology of varicocele, including the higher temperature of testis induced by poor venous drainage, the disorder of neuroendocrine system, the hypoxia of testis, the accumulation of metabolites in testis, the regurgitation of adrenal and renal metabolites, and the high levels of oxidative stress in left testis [25–27]. The Sertoli cell in varicocele is reported to be more sensitive than germ cells to changes in the testicular microenvironment [28] and the structure of the TJ between Sertoli cells is damaged in experimental varicocele in the rat testis [29, 30], implying that the Sertoli cell may be the primary altered intratubular site, leading to secondary spermatogenesis obstruction. In addition, the circulating antisperm antibodies can be detected in men with varicoceles, which imply that the structure of TJ (the primary composition of BTB) is disrupted probably [31]. Varicocele also can decrease T level in the left testicular tissue and serum [32]. Concurrently, the varicocele increases GnRH [33], luteinizing hormone (LH) [34], and FSH [35] levels and decreases inhibin B (INHB) levels [36] in the serum via its effect on the hypothalamic-pituitary-gonadal (HPG) axis. However, whether the varicocele affects cytokine and TJ protein levels in the testis remains unknown.

This study was designed to evaluate the left testis morphology, germ cell apoptosis, sperm count, the expression of Sertoli cell TJ proteins (Occludin, Claudin-11, and ZO-1), changes in cytokine (TGF-*β*3 and TNF-*α*) and T levels in the left testicular tissue, and GnRH, FSH, LH, T, INHB, and antisperm antibody (AsAb) serum levels. Meanwhile, we investigated whether and how MOP can promote spermatogenesis and repair the damaged left testis and TJ in the varicocele.

## 2. Materials and Methods

### 2.1. Experimental Materials

The reagents used in our study were as follows: rabbit anti-Occludin polyclonal antibody (Abcam Inc., Cambridge, MA, USA), rabbit anti-Claudin-11 polyclonal antibody (Abcam Inc.), goat anti-ZO-1 polyclonal antibody (Santa Cruz Biotechnology Inc., Dallas, TX, USA), mouse anti-*β*-actin monoclonal antibody (Dingguo Changsheng Biotechnology Co., Ltd., Beijing, China), HRP-conjugated goat anti-rabbit IgG (Beyotime Institute of Biotechnology, Shanghai, China), HRP-conjugated horse anti-goat IgG (Dingguo Changsheng Biotechnology Co., Ltd.), HRP-conjugated goat anti-mouse IgG (Dingguo Changsheng Biotechnology Co., Ltd.), bovine albumin (Beyotime Institute of Biotechnology), Ehanced BCA Protein Assay Kit (Beyotime Institute of Biotechnology), Radio Immunoprecipitation Lysis Buffer (RIPA, Beyotime Institute of Biotechnology), PMSF (Beyotime Institute of Biotechnology), prestained protein ladder (Thermo Fisher Scientific Inc., Waltham, MA, USA), polyvinylidene difluoride membrane (PVDF, Millipore Inc., Boston, MA, USA), Beyo ECL Star (Beyotime Institute of Biotechnology), 3,3-Diaminobenzidine Tertrahydrochloride (DAB, Beyotime Institute of Biotechnology), Easy Script one-step gDNA removal and cDNA synthesis super mix (TransGen Biotech Co., Ltd., Beijing, China), Trans Start Tip Top green qPCR super mix (TransGen Biotech Co., Ltd.), Colorimetric terminal deoxynucleotidyl transferase-mediated dUTP-biotin nick end labeling (TUNEL) Apoptosis Assay Kit (Beyotime Institute of Biotechnology), enzyme-linked immunosorbent assay (ELISA) Kit for Rat TGF-*β*3 (Westang Biotechnology Co., Ltd., Shanghai, China), ELISA Kit for Rat TNF-*α* (Westang Biotechnology Co., Ltd.), ELISA Kit for Rat FSH (Westang Biotechnology Co., Ltd.), ELISA Kit for Rat LH (Westang Biotechnology Co., Ltd.), ELISA Kit for Rat GnRH (Westang Biotechnology Co., Ltd.), ELISA Kit for Rat Inhibin B (Westang Biotechnology Co., Ltd.), ELISA Kit for Rat T (Westang Biotechnology Co., Ltd.), and Rat AsAb ELISA Kit (Mlbio Biotechnology Co., Ltd.).

### 2.2. Animal Samples

The rats used in the research were born and housed in the Laboratory Animal Center of Fujian Medical University. The experiments were conducted following the* Guidelines for the Care and Use of Laboratory Animals* approved by Fujian Medical University (Animal Approval Committee # SYXK-2012-0001).

Seventy-five male adolescent Sprague–Dawley rats (6-7 weeks old) were randomly and equally divided into five experimental groups: a sham group, a varicocele-induced model group, and three varicocele-induced groups treated with MOP 200 mg kg^−1^, 300 mg kg^−1^, and 400 mg kg^−1^, respectively. The experimental varicocele was established by partial ligation of the left kidney vein as previously described [37]. The rats in the sham group underwent the same procedures without ligation. Eight weeks after the operation, saline (2 mL) was given to rats in the varicocele-induced model group and different doses of MOP were given to the rats in the three administered groups by gavage daily for 4 weeks. The rats were weighted and anesthetized with 4–6 mg kg^−1^ chloral hydrate to check the left spermatic vein and left kidney, and those in the experimental varicocele groups with failed vascular dilation or left renal atrophy were excluded from the experiment.

### 2.3. *M. officinalis* Polysaccharide (MOP) Extracts

*M. officinalis* (Bajitian in Chinese), the dried rhizome of plant* Morinda officinalis F.C.How, *used in our experiment originates from Fujian Province in China. The plant name has been checked with http://www.theplantlist.org/. MOP was prepared by using the traditional method of water extraction and alcohol precipitation [38]. The dried and crushed* M. officinalis* was extracted 3 times (2 h each time) with ethyl alcohol at 90°C to remove the oligosaccharide and lipid components. The residue was then collected and extracted 3 times (2 h each time) with deionized water (three times the volume) at 100°C. The water extract was concentrated with a rotary evaporator at 50°C until the relative density reached 1.2 g mL^−1^. Next, the liquid extract was mixed with dehydrated ethanol (ethanol final concentration, 60%) and incubated overnight at 4°C in a refrigerator to obtain the crude polysaccharide. To remove the proteins, the polysaccharide was mixed with deionized water, Sevag reagent was added (chloroform :* n*-butyl alcohol = 1 : 1, one-third the volume) in a separating funnel, shaken, and allowed to set for 1 h. Then, the first stratification was collected. The whole protein removing process was repeated 3 times. The extract was mixed with dehydrated ethanol (ethanol final concentration, 60%) again, incubated overnight at 4°C to extract the polysaccharides without protein. Finally, the polysaccharides were washed with dehydrated ethanol, acetone, and diethyl ether in turn. The determination of polysaccharide percentage was performed with the anthrone-sulfuric acid method.

### 2.4. Hematoxylin-Eosin (H&E) Staining

H&E staining was conducted as previously described [39]. In brief, the animals were anesthetized with chloral hydrate and then perfused through the cardiac artery with saline and 10% formalin in 0.02 mol L^−1^ phosphate buffer solution in turn. Next, the left testis tissue was cut and fixed in 10% formalin for 24 h then embedded in paraffin. The coronal sections (5 *μ*m thick) were set on poly-l-lysine-coated slides, stained with H&E, and mounted with neutral balsam.

### 2.5. Epididymal Sperm Count

The sperm count of the left epididymis was conducted as previously described using a hemocytometer with some modifications [40]. In brief, the tail of the left epididymis was placed in 4 mL of preheated normal saline at 37°C in an tube, cut into pieces, and gently shaken 30 times. Next, the EP tube was placed into a 37°C incubator with an atmosphere of 5% CO_2_ for 10 min to let the sperm swim out. After being filtrated with a 200-mesh cell filter, 1 mL of epididymal sperm was diluted to 1 : 10 with normal saline. Finally, 10 *μ*L of the diluted sperm was added to each chamber of the hemocytometer, observed, and counted under light microscope (400x).

### 2.6. Terminal Deoxynucleotidyl Transferase-Mediated dUTP-Biotin Nick End Labeling (TUNEL) Assay

Apoptosis of the left testicular germ cells was assessed by TUNEL. The apoptotic cells were detected with a Colorimetric TUNEL Apoptosis Assay Kit according to the manufacturer's instruction. Briefly, the paraffin-embedded sections (5 *μ*m thick) were treated with 20 *μ*g mL^−1^ proteinase K (DNase free) after being deparaffinized and rehydrated at 37°C for 30 min and then washed with PBS three times. Subsequently, the sections were incubated with biotin-labeled UTP and terminal deoxynucleotidyl transferase (TdT) at 37°C for 60 min. After being washed with PBS three times, the sections were treated with streptavidin-HRP at room temperature for 30 min followed by coloration with diaminobenzidine (DAB) and hematoxylin. Finally, the slides were mounted with neutral balsam. The percentages of apoptotic cells and tubules were calculated as previously described [41]. Left testes from 6 animals in each group were used and 5 to 10 fields were randomly chosen from each testis. The percentage of apoptotic cells was determined by counting TUNEL-positive cells out of 1,000 germ cells from 15–20 seminiferous tubules in coronal sections of each testis. The percentage of apoptotic tubules was determined by counting TUNEL-positive tubules in 50 seminiferous tubules in coronal sections of each testis.

### 2.7. Western Blot Analysis

Western blot was performed as previously described with some modifications [42]. The total protein was extracted from the left testis tissue using RIPA lysis buffer with 1 mM PMSF, and the protein concentration was measured with an enhanced BCA protein assay. Denatured protein (80 *μ*g) was separated by SDS-PAGE gel electrophoresis accompanied with 210 kDa to 10 kDa weight prestained protein ladder and transferred onto polyvinylidene difluoride membrane (PVDF) for immunodetection using primary antibodies against Occludin (1 : 200), Claudin-11 (1 : 250), ZO-1 (1 : 100), and *β*-actin (1 : 1000). The PVDF membrane was then incubated with the corresponding secondary antibody, including HRP-conjugated goat anti-mouse IgG (1 : 1000), HRP-conjugated goat anti-rabbit IgG (1 : 1000), and HRP-conjugated horse anti-goat IgG (1 : 1000). Finally, the signal was detected using enhanced chemiluminescence (ECL) and the images were acquired with a biological imaging system.

### 2.8. Transmission Electron Microscopy

Fresh testis tissue was cut into thin slices and placed in 3% glutaraldehyde-1.5% paraformaldehyde-0.1 mol L^−1^ PBS (pH 7.2) for more than 2 h and then examined with a transmission electron microscope in the electron microscopy laboratory of Fujian Medical University.

### 2.9. ELISA

Blood was drawn from the rat heart and placed at 4°C for 12 h. Next, the blood was centrifuged at 5000 r/min for 10 min and the serum was harvested from the top layer and then stored at −80°C for ELISA. As for the left testicular tissue, the total protein was extracted from the left testis tissue using PBS and the protein concentration was measured with an enhanced BCA protein assay. Other procedures were performed according to the manufacturer's instructions.

### 2.10. Quantitative Real-Time PCR Analysis (qPCR)

Total RNA was extracted from the left testis tissue using TRIzol reagent. The first strand cDNA was synthesized by Applied Biosystems 9700 PCR System using 1 *μ*g total RNA in the light of the instruction. The levels of mRNA were assessed by quantitative real-time PCR technology using the ABI7500 PCR machine and SYBR Green according to the manufacturer's instruction. The real-time PCR primers used are presented in Table 1.

### 2.11. Statistical Analysis

All experiments were performed at least three times independently. The data were analyzed with SPSS version 21.0 (SPSS Inc., Chicago, IL, USA) and expressed as mean ± standard deviation (SD). The one-way ANOVA test was used to analyze the significant difference between groups and values of *P* < 0.05 were considered statistically significant.

## 3. Results

### 3.1. Left Testis Morphology

**Figure 1:**
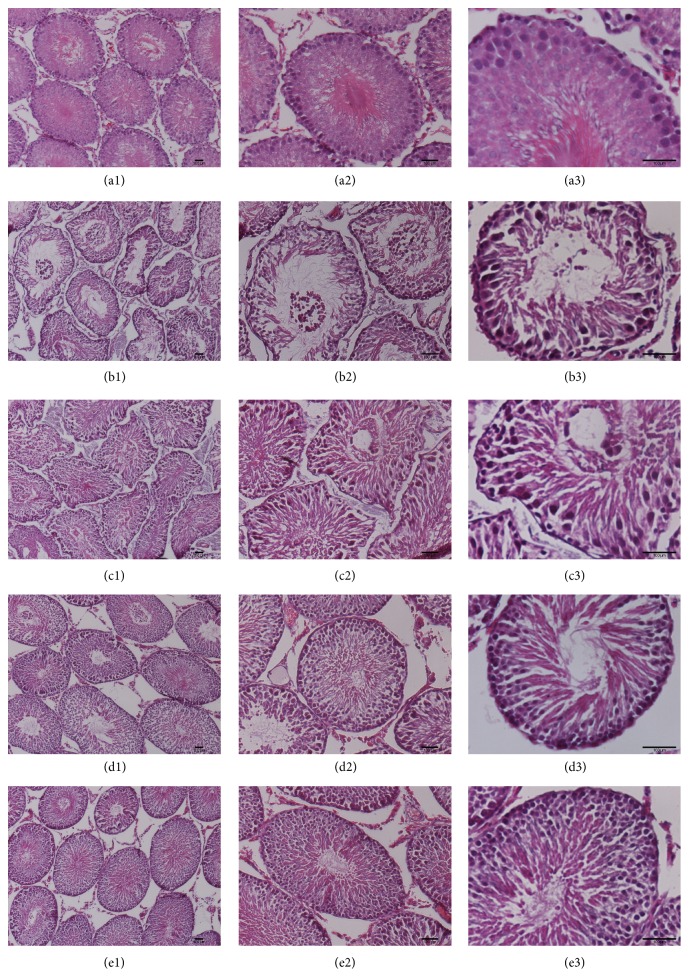
Changes in the left testis morphology. Three images at different magnifications are presented for each group. The normal testis morphology is shown in (a1), (a2), and (a3). (b1), (b2), and (b3) present the damaged spermatogenic epithelium caused by experimental varicocele, which is characterized by a thinner spermatogenic epithelium, less germ cells and mature sperms, atrophic seminiferous tubules, disorganized germ cells at different phases, and shedding of the immature germ cells in the adluminal compartment. In (c1), (c2), and (c3); (d1), (d2), and (d3); and (e1), (e2), and (e3), different dosages of MOP repaired the damaged structure of the spermatogenic epithelium, and the 300 mg kg^−1^ and 400 mg kg^−1^ doses of MOP presented a better therapeutic effect.

H&E staining showed that the experimental varicocele resulted in the destruction of the spermatogenic epithelium. The varicocele-induced rats presented with a thinner spermatogenic epithelium, less germ cells and mature sperms, atrophic seminiferous tubules, disorganized germ cells at different phases, shedding of the immature germ cells in the adluminal compartment (Figures 1(b1), 1(b2), and 1(b3)) in comparison to the control animals (Figures 1(a1), 1(a2), and 1(a3)). Meanwhile, both the 300 mg kg^−1^ and the 400 mg kg^−1^ MOP doses repaired the damaged spermatogenic epithelium as shown by the increased thickness of the spermatogenic epithelium and increased number of germ cells and mature sperms, as well as the decreased number of deciduous immature germ cells as shown in Figures 1(d1), 1(d2), 1(d3), 1(e1), 1(e2), and 1(e3) compared with Figures 1(b1), 1(b2), and 1(b3). However, the structure did not completely recover to the normal level when compared to the control rats (Figures 1(a1), 1(a2), and 1(a3)). The repairing effect of 200 mg kg^−1^ MOP dose (Figures 1(c1), 1(c2), and 1(c3)) was not as good as the 300 mg kg^−1^ and 400 mg kg^−1^ dose, which meant the pharmacological function of MOP showed a dose-dependent effect.

### 3.2. Sperm Count in the Left Cauda Epididymis and Left Testis/Epididymis Index

As shown in Table 2, the number of sperms in the tail of the left epididymis, the left testis index, and the left epididymis index of the varicocele-induced rats was lower than that of the sham group and MOP increased the quantity of sperms, testis index, and epididymis index.

### 3.3. Germ Cell Apoptosis

**Figure 2:**
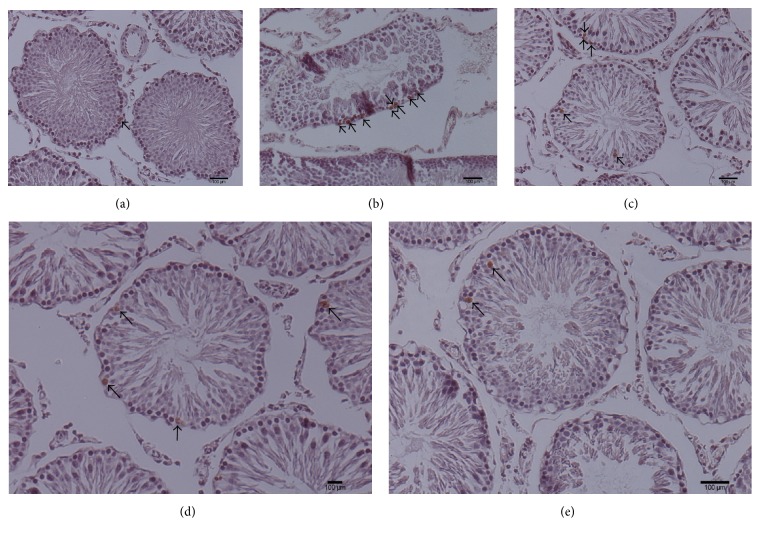
Germ cell apoptosis in the left testis. (a), (b), (c), (d), and (e) are the representative images of the sham group, the model group, and three varicocele-induced groups treated with MOP 200 mg kg^−1^, 300 mg kg^−1^, and 400 mg kg^−1^, respectively, the nucleus of TUNEL-active cells are stained brown (see black arrowheads). The experimental varicocele increased the number of apoptotic cells, while MOP decreased germ cell apoptosis.

As shown in Figure 2 and Table 2, percentages of both apoptotic cells and apoptotic tubules in the experimental varicocele model rats were higher than those of the sham animals, while MOP decreased the percentages of apoptotic cells and tubules in the left testis.

### 3.4. Changes in TJ Structure

**Figure 3:**
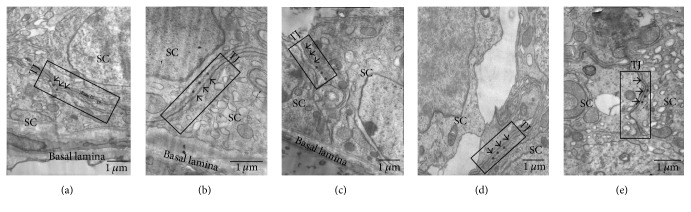
Changes in the TJ structure in the left testis. The TJ strand (see black arrowheads) is shown in the black boxed area in (a), which coexists with the basal ES. The basal ES present on both sides of the two adjacent Sertoli cell (SC) is typified by actin filament bundles existing between the endoplasmic reticulum (ER, see black asterisks) and TJ strand (see black arrowheads). In (b), the TJ of the varicocele model rats is injured, as shown by the loss of the basal ES, the disintegrated actin filament bundles, and the disorganized ER; see black asterisks in (b) and (a); the TJ strand is also broken, shown as the widened intercellular space between the two apposing Sertoli cells (see black arrowheads in (b)). In (c), (d), and (e), the structure of TJ in the MOP administered animals is repaired to varying degrees, but not completely normal. These results are representative of three rats from each group.

The varicocele-induced damage to the TJ was observed by transmission electron microscopy. As shown in Figure 3(a), the TJ strand (see black arrowheads in boxed area) coexisted with actin filament bundles and the neighboring endoplasmic reticulum (ER, see black asterisks). The actin filament bundles were sandwiched between the endoplasmic reticulum (ER) and the TJ strand (black boxed area), which formed the typical structure of basal extracellular specialization (ES) detected on both sides of adjacent Sertoli cells in the rat testis. In Figure 3(b), TJ (see black arrowheads in boxed area) is damaged, as shown by the increased intercellular space and fuzzy TJ strand. Meanwhile, the coexisting basal ES is also disturbed, the actin filament bundles and the neighboring endoplasmic reticulum (ER) become severely disordered, defragmented, or even lost (see black asterisks in the boxed area in Figure 3(b)). As shown in Figures 3(c), 3(d), and 3(e), the structure of TJ in the MOP administered groups was repaired to varying degrees but did not completely resemble the normal TJ. For example, the TJ strand became clear (see black arrowheads in the boxed area in Figure 3(d)), but the actin filament bundles and endoplasmic reticulum (ER) remained disorganized and lost (see black asterisks in the boxed area in Figure 3(d)).

### 3.5. Changes in TJ Protein Expression

**Figure 4:**
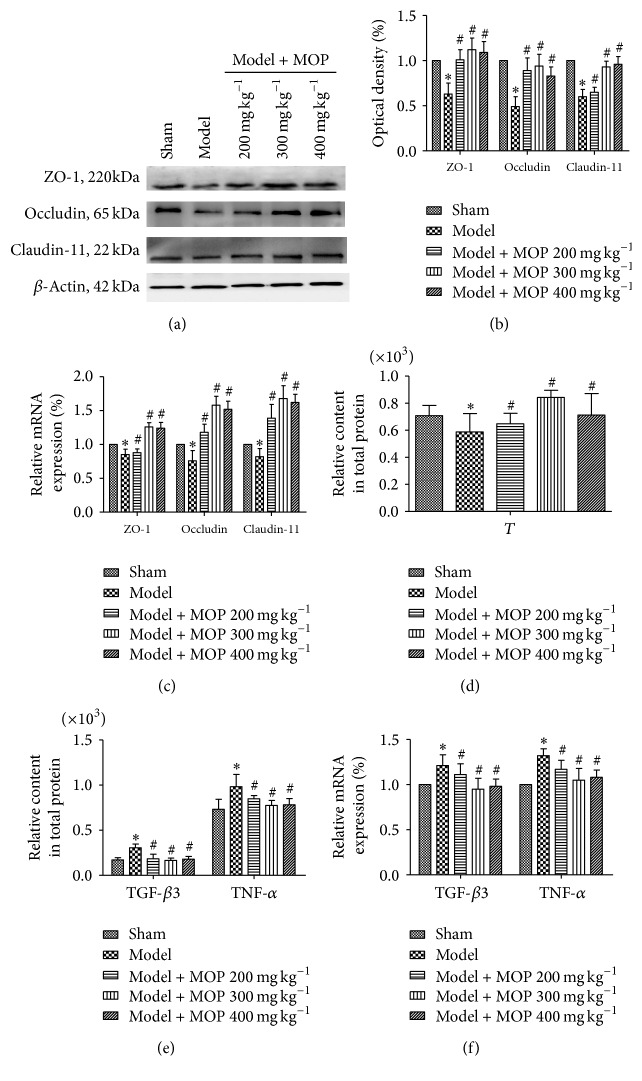
Changes in TJ protein expression, the level of cytokines, TGF-*β*3 and TNF-*α*, and the level of testosterone (T) in the left testicular tissue. (a) and (b) Western blot analysis shows that the expression of Occludin, Claudin-11, and ZO-1 in the varicocele model group was lower than that in the sham group. A significant increase in Occludin, Claudin-11, and ZO-1 expression is observed in the MOP administered animals compared to that in the varicocele model group. (c) qPCR results for Occludin, Claudin-11, and ZO-1 mRNA levels were similar to the western blot results. (d) ELISA shows that a decrease in T level in left testicular tissue is detected in the varicocele model group when compared to that in the sham group and each MOP administered group showed a higher T level compared to the varicocele model group. (e) An increase in the levels of TGF-*β*3 and TNF-*α* in the varicocele model group compared to the sham group and a decrease in each administered group compared to the model group were observed by ELISA. (f) TGF-*β*3 and TNF-*α* mRNA levels were measured by qPCR, the results were similar to the ELISA results. The data are presented as mean ± SD, bars indicate SD, *n* = 6, # compared to the model group (*P* < 0.05), and *∗* compared to the sham group (*P* < 0.05).

Figures 4(a) and 4(b) show that experimental varicocele decreased the expression of TJ proteins, Occludin, Claudin-11, and ZO-1, while MOP increased their expression.  Figure 4(c) shows that Occludin, Claudin-11, and ZO-1 mRNA expression was similar to their protein expression assessed by western blot (*P* < 0.05).

### 3.6. Changes in Cytokine Levels in Left Testis

The relative expression of TGF-*β*3 and TNF-*α* and their relative mRNA levels in the left testis tissue were detected by ELISA and qPCR, respectively. As shown in Figure 4(e), levels of TGF-*β*3 and TNF-*α* in the model group were higher than those of the sham group (*P* < 0.05). Additionally, TGF-*β*3 and TNF-*α* levels in each of the MOP groups were lower compared to those of the model group (*P* < 0.05). As shown in Figure 4(f), the qPCR results were similar to those obtained by ELISA (*P* < 0.05), indicating that experimental varicocele can increase the expression of TGF-*β*3 and TNF-*α* in the left testis, while MOP can lower the cytokine levels.

### 3.7. Changes in T Level in the Left Testicular Tissue and Serum GnRH, FSH, LH, INHB, T, and AsAb Levels

T levels in the left testicular tissue, the related serum sex hormone levels, and the concentration of AsAb in the serum were detected by ELISA. As shown in Figure 4(d), T levels in the left testicular tissue in the model group was lower than that in the sham group (*P* < 0.05). Meanwhile, T levels in each MOP administered group increased when compared to those in the model group (*P* < 0.05).

Table 3 indicates that serum levels of GnRH, FSH, and LH in the model group are higher than those in the sham group (*P* < 0.05), and those in each MOP administered group are lower than that in the model group (*P* < 0.05). The serum INHB and T levels of the model group were lower than those of the sham group (*P* < 0.05), and those of each MOP administered group were higher than those of the model group (*P* < 0.05). A significant increase in serum AsAb levels was detected in the model group when compared to the sham group (*P* < 0.05) and a slight decrease in AsAb levels was observed in each MOP administered group when compared to the model group (*P* < 0.05).

## 4. Discussion

### 4.1. MOP Promotes Spermatogenesis and Regulates the Levels of Serum Sex Hormones in Experimental Varicocele Rats

Varicocele is a common urological disease. However, why it induces damage to the testis and results in man infertility remains controversial [43]. The results of epididymal sperm count, morphological analysis, and TUNEL assay indicated that MOP not only promoted spermatogenesis but also decreased germ cell apoptosis. Nevertheless, the mechanism underlying such repair effect on experimental varicocele rat testis is complicated and yet to be discovered. Many traditional Chinese medicines used to treat reproductive system diseases present antioxidant and antiestrogenic properties [44] and enhance the ability of sexual glands, like increasing T levels [43]. They also present a function similar to that of GnRH, which may also explain the pharmacological function of MOP on repairing the varicocele-impaired male reproductive system.

As mentioned previously, varicocele disturbs hormone levels via its effects on Leydig cells, Sertoli cells, and the hypothalamic-pituitary-gonadal (HPG) axis (decreasing T and INHB levels, while increasing GnRH, FSH, and LH levels in the serum) [32–35]. In our study, we determined that varicocele increased the levels of GnRH, FSH, and LH, while MOP abrogated this effect, indicating that MOP can regulate the abnormal serum sex hormone levels to reduce them back to the normal levels in varicocele. Secondly, varicocele decreased serum levels of T and INHB. This effect was reversed by MOP, indicating that varicocele altered Sertoli and Leydig cell functions and that MOP repaired such damage, as shown by the increased corresponding sex hormones levels. In brief, our results illustrated that MOP could regulate the abnormal serum levels of sex hormones induced by varicocele to bring them back to the normal level, thereby improving the male reproductive system at the integral level. Thus, MOP may influence the hypothalamic-pituitary-gonadal (HPG) axis and enhance the ability of sexual glands.

### 4.2. MOP Repairs TJ in Experimental Varicocele Rat Testis

In general, the BTB is largely formed by TJ between paratactic Sertoli cells adjacent to the basement membrane, which establishes an immunological barrier by separating the germ cells from the systemic circulation. Meanwhile, TJ has to timely open and close to let the preleptotene/leptotene spermatocytes come across during stages VIII–XI of the spermatogenic cycle in rats in spite of being one of the tightest barriers in mammals. To date, three classes of TJ transmembrane proteins and one of associated proteins (linking transmembrane proteins to the cytoskeleton) have been confirmed in the testis, including Occludin, Claudin, junctional adhesion molecules (JAM), and zonula occludens (ZO) [45, 46]. TJ proteins in Sertoli cells are more sensitive to reproductive toxicity, possibly due to the unique dynamics of TJ [23, 28, 29]. Meanwhile, destruction of junctional complex between Sertoli cells or Sertoli and germ cells may result in spermatogenesis obstruction [47]. In our study, varicocele disrupted the structure and function of TJ, as shown by the increased level of AsAb in the serum, the low expression of tight junction proteins, an increase in the intercellular space, the fuzzy TJ strand, and the disorganized basal ES. MOP could increase the level of Occludin, Claudin-11, and ZO-1, decrease the level of AsAb in the serum, and repair the damaged TJ structure and function.

The expression of TJ proteins can be regulated by hormones (e.g., T and FSH) and cytokines (e.g., TGF-*β*3, TNF-*α*, and IL-6) [11, 13–17]. For example, TGF-*β*3 is involved in the destruction of Sertoli cell TJ dynamics, possibly via its decreasing effects on the expression of Occludin, Claudin-11, and ZO-1 [13]. TNF-*α* can downregulate the steady-state protein level of Occludin and ZO-1 in the rat testis [48]. In our study, varicocele increased TGF-*β*3 and TNF-*α* levels in the left testicular tissue. However, MOP can decrease TGF-*β*3 and TNF-*α* levels. As a result, we believe that TGF-*β*3 and TNF-*α* might be involved in the downregulation of TJ proteins, Occludin, Claudin-11, and ZO-1, and in the damage of the TJ structure that occurred in experimental varicocele. Furthermore, MOP can up-regulate the expression of Occludin, Claudin-11, and ZO-1 by reducing TGF-*β*3 and TNF-*α* expression. We also observed a decrease in the T level in the left testicular tissue of the varicocele group compared to that of the sham group. MOP increased the T level, which also contributed to the induction of TJ protein expression.

In summary, MOP can repair the damaged TJ structure and function and upregulate the expression of TJ proteins (Occludin, Claudin-11, and ZO-1), which were downregulated by experimental varicocele in the rat testis, probably via decreasing the level of cytokines (TGF-*β*3 and TNF-*α*) and increasing the T level in the left testicular tissue. Obviously, the changes in TJ protein (Occludin, Claudin-11, and ZO-1) expression caused by varicocele and MOP are the result of complicated biological events. They are not limited to the regulation of the cytokines and hormones analyzed in this study. The specific mechanism by which MOP decreases the level of cytokines (TGF-*β*3 and TNF-*α*) and increases the T level in varicocele testicular tissue remains unknown and further studies are warranted to elucidate this mechanism.

### 4.3. Mechanism by Which MOP Affects TJ and Promotes Spermatogenesis in Experimental Varicocele Rats

The mechanism underlying the effects of MOP on the TJ of experimental varicocele rat testis may relate to its bioactivities such as its immunomodulatory, anticancer, and antioxidant properties [49–51]. First, MOP antioxidant activity may contribute to repairing the damaged TJ structure and the testis tissue in experimental varicocele rats. Secondly, MOP regulates the level of sex hormones probably via its effect on the hypothalamic-pituitary-gonadal (HPG) axis, Sertoli cells, and Leydig cells, meaning that MOP can regulate the abnormal hormone levels, thereby reducing the damage caused by varicocele. Thirdly, MOP-induced downregulation of TGF-*β*3 and TNF-*α* may relate to the fact that MOP can decrease the inflammatory reaction caused by testicular hypoxia and the increased testicular temperature in the varicocele rat testis. Lastly, the curative effect of MOP on varicocele testis may be associated with its function on stimulating blood circulation and removing toxic metabolites in blood stasis.

There are various approaches to treating varicocele in clinical practice, like open surgical and percutaneous embolization. However, all these treatments show varying degrees of complications and recurrence. Meanwhile, the effectiveness of varicocele treatments in improving male fertility remains highly controversial, especially for adolescents and men with subclinical varicocele [52]. In China, many infertile couples take traditional Chinese herbal medicine to treat infertility, as it is thought to be more convenient, safer, and more effective [43]. Our goal was to identify a traditional Chinese herbal medicine extract that can be used in combination with varicocelectomy to treat male infertility caused by varicocele, to help the patients get more effective treatment, and to promote fertility. However, traditional Chinese herbs are composed of many ingredients, which may result in different degrees of hepatotoxicity and renal toxicity when they are used clinically. Therefore, we needed to identify the most effective ingredient in* M. officinalis* to promote fertility and avoid its toxicity. Combining previous studies from our laboratory [4, 5] with other related studies, we speculated that MOP may be the most effective component and the present study confirmed this hypothesis. Indeed, the results showed that MOP can repair the impaired male reproductive system caused by varicocele. It is a very potent Chinese traditional medicine for male infertility with a considerable clinical value. However, additional experiments are warranted to understand the mechanism underlying the effects of MOP in the regulation of the male reproductive system. In addition, we find the most effective daily dose of MOP on experimental varicocele rat is 300 mg kg^−1^ in our study, according to the clinical use of* M. officinalis* at present, this dose of MOP is safe for human. Because if the daily dose 300 mg kg^−1^ of MOP for rat is extrapolated to the human equivalent dose (HED) by a conversion based on body weight, the equivalent daily dose of MOP for human is about 50 mg kg^−1^. In clinical practice, the treatment dosage of* M. officinalis* is about 15–30 g per day for human when it is used to tonify the kidney and strengthen yang in traditional Chinese medicine clinical practice. The polysaccharides account for about 20% on average of the total weight in* M. Officinalis *[2, 3], which means the corresponding treatment dosage of MOP for human is 3–6 g per day; if the average weight of human is 60 kg, then the daily dose of MOP for human is about 50–100 mg kg^−1^. Therefore, we consider that the effective daily dose 300 mg kg^−1^ of MOP in rat model is safe for human according to the use of* M. officinalis* in clinical practice at present.

## Figures and Tables

**Table 1:** Quantitative real-time PCR primer sequences.

Gene	Primer sequence
Occludin	F: ATGCTGGTTGCTGGAGAAGTG
	R: GGAGACAGGAAACGGATGGTATT
Claudin-11	F: GGCGTTCCATTGTTGTTGAT
	R: GTCTCTACGAGGCTTCCATTGT
ZO-1	F: GAGGCTTCAGAACGAGGCTAT
	R: TGCTTCGGCTCAGATGACTTA
TNF-*α*	F: GGCAGGTCTACTTTGGAGTCATTG
	R: TTTCTGAGCATCGTAGTTGTTGGA
TGF-*β*3	F: CGCTACATAGGTGGCAAGAAT
	R: CAGTCGGTGTGGAGGAATCA
GAPDH	F: ACGGCAAGTTCAACGGCACAG
	R: GAAGACGCCAGTAGACTCCACGAC

**Table 2:** Effects of MOP on related parameters of the left testes of experimental varicocele model rats (*n* = 6).

	Sham	Model	Model + MOP	Model + MOP	Model + MOP
			200 mg kg^−1^	300 mg kg^−1^	400 mg kg^−1^
Testis index (weight/BW%)	0.41 ± 0.021	0.33 ± 0.012^*∗*^	0.35 ± 0.015^#^	0.38 ± 0.011^#^	0.37 ± 0.031^#^
Epididymis index (weight/BW%)	0.15 ± 0.0075	0.12 ± 0.0071^*∗*^	0.13 ± 0.0081^#^	0.14 ± 0.0049^#^	0.14 ± 0.0051^#^
Sperm count (×10^6^ mL^−1^)	45.03 ± 3.46	30.63 ± 3.47^*∗*^	31.7 ± 4.11^#^	35.89 ± 4.21^#^	36.27 ± 3.62^#^
Apoptotic cell percentage (%)	1.02 ± 0.19	5.02 ± 0.41^*∗*^	3.62 ± 0.33^#^	2.77 ± 0.23^#^	2.51 ± 0.28^#^
Apoptotic tubule percentage (%)	7.67 ± 1.51	26.33 ± 2.94^*∗*^	19.72 ± 2.12^#^	16.33 ± 1.63^#^	15.83 ± 1.64^#^

BW, body weight; MOP, *Morinda officinalis* Polysaccharide. The data are presented as mean ± SD; *∗* compared to the sham group (*P* < 0.05); # compared to the varicocele model group (*P* < 0.05).

**Table 3:** Changes in the levels of hormone and antisperm antibody in serum (*n* = 6).

	Sham	Model	Model + MOP	Model + MOP	Model + MOP
			200 mg kg^−1^	300 mg kg^−1^	400 mg kg^−1^
Gonadotropin-releasing hormone (GnRH) (pg mL^−1^)	648.84 ± 1.44	686.22 ± 2.67^*∗*^	665.81 ± 1.67^#^	653.66 ± 2.10^#^	652.95 ± 1.53^#^
Follicle-stimulating hormone (FSH) (ng mL^−1^)	12.23 ± 0.22	13.51 ± 0.31^*∗*^	13.15 ± 0.16^#^	12.82 ± 0.24^#^	12.77 ± 0.12^#^
Luteinizing hormone (LH) (ng mL^−1^)	6.095 ± 0.19	6.67 ± 0.33^*∗*^	6.45 ± 0.21^#^	6.27 ± 0.19^#^	6.16 ± 0.30^#^
Inhibin B (INHB) (pg mL^−1^)	22.77 ± 0.27	20.58 ± 0.20^*∗*^	20.90 ± 0.10^#^	21.048 ± 0.17^#^	21.087 ± 0.22^#^
Testosterone (T) (ng mL^−1^)	4.56 ± 0.15	3.57 ± 0.18^*∗*^	3.78 ± 0.13^#^	3.85 ± 0.096^#^	3.89 ± 0.084^#^
Antisperm antibody (AsAb) (pg mL^−1^)	2.42 ± 0.08	66.97 ± 0.27^*∗*^	64.95 ± 0.14^#^	63.13 ± 0.19^#^	62.67 ± 0.21^#^

MOP, *Morinda officinalis* Polysaccharide. The data are presented as mean ± SD; *∗* compared to the sham group (*P* < 0.05); # compared to the varicocele model group (*P* < 0.05).
